# Postural Control and Stress Exposure in Young Men: Changes in Cortisol Awakening Response and Blood Lactate

**DOI:** 10.3390/ijerph17197222

**Published:** 2020-10-02

**Authors:** Marinella Coco, Andrea Buscemi, Emanuele Pennisi, Paolo Cavallari, Giacomo Papotto, Giulio Maria Federico Papotto, Vincenzo Perciavalle, Donatella Di Corrado, Valentina Perciavalle

**Affiliations:** 1 Department of Biomedical and Biotechnological Sciences, University of Catania, 95123 Catania, Italy; 2 Motor Activity Research Center (CRAM), University of Catania, 95123 Catania, Italy; 3 Horus Social Cooperative, Department of Research, 97100 Ragusa, Italy; andreabuscemi@virgilio.it; 4 Department of Research, Italian Center Studies of Osteopathy, 95100 Catania, Italy; 5 Department of Educational Sciences, University of Catania, 95100 Catania, Italy; alfio.pennisi@unict.it (E.P.); valentinaperciavalle@hotmail.it (V.P.); 6 Department of Pathophysiology and Transplantation, Human Physiology Section, University of Milan, 20122 Milan, Italy; paolo.cavallari@unimi.it; 7 University Hospital “Policlinico G. Rodolico-San Marco”, University of Catania, 95123 Catania, Italy; giacomopapotto@gmail.com; 8 “Bianchi-Melacrino-Morelli” Hospital of Reggio Calabria, 89124 Reggio Calabria, Italy; g.pap8@hotmail.it; 9 Department of Human and Social Sciences, School of Sport Sciences, Kore University, 94100 Enna, Italy; didinawoody@gmail.com (D.D.C.); perciava@libero.it (V.P.)

**Keywords:** stress, postural control, salivary cortisol, blood lactate, gender, laterality

## Abstract

Background: It has recently been noticed that the quantity of stress affects postural stability in young women. The study was conducted with the goal of investigating whether increased stress may damagingly effect posture control in 90 young men (71 right-handed and 19 left-handed) while maintaining an upright bipedal posture, while keeping their eyes open or closed. Perceived Stress Scale (PSS) was administered and changes in free cortisol levels were monitored (Cortisol Awakening Response, CAR) in order to evaluate the amount of stress present during awakening, while the Profile of Mood States (POMS) was used to estimate distress on the whole. Posture control was evaluated with the use of a force platform, which, while computing a confidence ellipse area of 95%, was engaged by the Center of Pressure through five stability stations and was sustained for a minimum of 52 s, with and without visual input. Another goal of the experiment was to find out whether or not cortisol increases in CAR were linked with rises of blood lactate levels. Results: CAR, PSS and POMS were found to be extensively related. Furthermore, it has been observed that increases in salivary cortisol in CAR are associated with small but significant increases in blood lactate levels. As expected, stress levels did affect postural stability. Conclusions: The results of the present study confirm that the level of stress can influence postural stability, and that this influence is principally obvious when visual information is not used in postural control.

## 1. Introduction

The ability to monitor the standing position is essential for walking and starting a quick, voluntary movement. This capability is frequently observed by examining the variants of the center of pressure (CoP). Many variables can influence the control of posture, such as hour of the day [1,2,3], level of fatigue [4], or age [5,6,7,8].

Mood directly influences mental and physical condition, varying from sadness and apprehension, to compulsion and stress [9], and physiologic responses to stress are significant aspects of health and disease [10]. The effects caused by stress can be both positive and negative, and are related to the frequency, intensity and duration of the stress [11]. High levels of stress are concomitant to the deterioration of executive functions, abstract reasoning, processing speeds and visual-spatial memory [10,11,12,13,14]. Moreover, it has been observed that mood state can affect control of balance [15] and anticipatory postural adjustments [16].

It has been observed that the amount of stress can influence postural stability in young women [17]. The present research was carried out to assess whether high levels of stress and emotionally adverse experiences may damagingly affect the control of posture in young men during quiet standing maintained with and without visual input. The aim was to identify possible gender differences in the influence exerted by stress on postural control, by comparing data observed in the present study carried out on males with data acquired from a previous study carried out on females [17]. Moreover, the study also investigated the possible role of laterality. The task consisted of quiet standing under five conditions selected from those recommended by Kirby et al. [18]. Three of these postures had the two feet on an identical level on the sagittal plane, but at a different distance apart; while, in the other postures one of the two feet (the right foot in the fourth position, left in the fifth position) was 10 cm forward.

The awareness of stress of the participants was evaluated with the Perceived Stress Scale (PSS), developed by Sheldon Cohen and coworkers [19], whereas POMS (Profile of Mood States) was administered to measure general mood disturbance. As a second measure of stress, we examined the Cortisol Awakening Response (CAR), i.e., a quick rise of free cortisol levels, in humans, happening in the 30 min after awakening and then returning to baseline values one hour later [20]. It has been observed that chronic stress plays a significant role on the CAR (for reviews, see [21,22]); many studies have been conducted on the relationship between CAR and cognitive performance but, to date, it has not been possible to draw definitive conclusions (for reviews, see [23]). For measuring the CAR, saliva samples were taken from the participants in this study.

Finally, since it was found that during a mentally and physically demanding exercise cortisol increases were associated with blood lactate rises [24,25,26,27], we also wanted to see if the salivary cortisol increases in CAR were associated with rises of blood lactate levels.

## 2. Materials and Methods

### 2.1. Participants

Ninety healthy young men, students at the University of Catania, participated in this study. Only male students were registered, as gender variations in brain structures control the activity of the HPA axis, as well as the levels of corticosteroid binding globulin that affect the HPA axis (for a review, see [28]). Participants were selected on the basis of close anthropometric characteristics; in fact, the mean age was 23.5 years (±2.05), the mean height was 172.5 cm (±3.83), mean weight was 74.7 kg (±4.07) and the mean Body Mass Index was 25.1 (±0.55). Seventy-one subjects were right-handed and nineteen left-handed. The Edinburgh Handedness Inventory [29] recognized laterality.

The protocol of the study was approved by the Ethical committee of the University of Milan (number 15/16). Participants were then informed of the ethics and signed an approved consent form (number 15/16).

### 2.2. Stress Measurement

The awareness of stress of participants was evaluated with the 10-item version of PSS [19], which holds 10 questions rated on a five-point Likert scale. Scores can vary from 0 to 40, with higher scores indicating larger individual distress. The PSS is designed to quantify the extent to which participants felt overwhelmed by stressful events over the last 30 days on a scale from 0 (never) to 4 (very often). The mean score for a normal population is 28.0 ± 8.71 [30].

McNair et al.’s 30-item version of the Profile of Mood States (POMS) [31] was used to analyze disturbances in the moods of participants. Participants evaluated each article on a five-point Likert scale ranging between “Not at all” to “Extremely”. Articles were created with the intent of producing six different subscales: vigor-activity (V), tension-anxiety (T), anger-hostility (A), fatigue-inertia (F), depression-dejection (D) and confusion-bewilderment (C). The raw values of each subscale were converted to a T-score by using the formula: T = 50 + 10 (n − m)/s, where n = raw value; m = mean; s = standard deviation. In this scenario, the raw data were turned into a standardized scale with a mean value for the single subscale of 50 ± 10 [32]. The T-scores of the dissimilar subscales were combined to obtain a total measure of distress marked Total Mood Disturbance (TMD = T + D + A − V + F + C). While the single subscales evaluate variations in specific aspects of mood, TMD denotes a single, global assessment of mood states.

### 2.3. Salivary Cortisol Assay

Participants were instructed to wake up between 6:00 h and 8:00 h on two consecutive days. This choice derives from the observation that the CAR, in terms of cortisol levels at each time point and the total amount of cortisol secreted during the measurement period, is stable across two consecutive mornings of measurement [33]. Subjects with differences of more than 15% between the two days were not included in the study. Samples of saliva were gathered on both days, at awakening (sample 1), and 15 min (sample 2), 30 min (sample 3), 45 min (sample 4) and 60 min (sample 5) thereafter, to obtain a total of five samples per day, and consequently ten samples for each participant, who had to stay in bed until all saliva samples were collected. Collection was carried out with a tampon that the participants had chewed for about a minute. For each participant, 10 sterile containers were marked beforehand with the identification code of the subject, the date, and the set time to repeat the sampling. The containers were frozen at −70° C until assay. Salivary levels of free cortisol were measured with a radioimmunoassay technique as described below [17,34].

Levels of salivary cortisol were measured using a Radioimmunoassay Cortisol Test (RIA CT from RADIM SpA, Rome, Italy). The procedure is founded on the competition between radiolabeled antigens and unlabeled antigens for antibody binding sites. As the concentration of unlabeled antigen is increased, antibody-bound radiolabeled antigen is released, and the radioactivity of the free antigens is measured in the supernatant using a gamma counter.

### 2.4. Blood Lactate

Levels of blood lactate were measured by each participant every time he took a saliva sample. The subject was trained to use a “Lactate Pro 2”, a portable lactate analyzer (Arkray Inc, Kyoto, Japan), which has proven to be highly reliable [35].

### 2.5. Balance Performance

**Figure 1:**
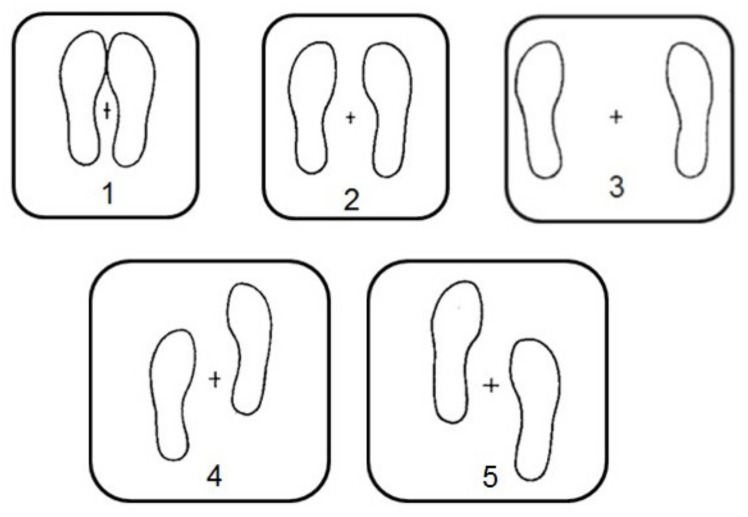
Positions used in the current experiments. (**1**) feet together; (**2**) feet 15 cm apart; (**3**) feet 30 cm apart; (**4**) right foot forward 10 cm; (**5**) left foot forward 10 cm (cfr. Coco et al., [17]).

The postural control measurements were carried out on the second day of collection of saliva samples. A Model OR-6-7-1000 force platform (Advanced Mechanical Technology Incorporated, Newton, MA, USA) was used to assess the variables of CoP. As shown in Figure 1, CoP measures were carried out over the course of the subsequent balance positions (cfr. [17]); (1) feet together; (2) feet 15 cm apart; (3) feet 30 cm apart; (4) right foot forward 10 cm; (5) left foot forward 10 cm (Figure 1). All 5 positions were maintained for 52 s with eyes open, and 52 s with eyes closed.

To avoid circadian or circannual influences on stability of posture [1], the trials were conducted between 10.00 AM and 1.00 PM and between January and February 2020. The participants were barefoot throughout the experiments. The AMTI force platform instantly evaluates the force components with respect to the x (medio–lateral, ML); y (antero–posterior, AP); and z (vertical, V) axes, and the moment components about the *x*-, *y*-, *z*-axes. The AMTI MiniAmp MSA-6 strain gauge amplifier system amplified the signals from the AMTI force platform and then digitized them at a 100 Hz sampling rate, using a 1401 data acquisition unit (Cambridge Electronic Design, Cambridge, UK). MATLAB software (The MathWorks Inc., Natick, MA, USA) was utilized to improve routines to analyze the area equivalent to 95% of the area designated by the CoP trajectory (A95), because preceding research has shown that this is the most efficient index of postural stability [36,37].

### 2.6. Data Analysis

The data were presented as a mean ± standard deviation (SD). In a two-day period salivary cortisol levels were averaged to acquire a single mean level of salivary cortisol, for every time point.

The rise in cortisol was considered as the outstanding factor between the single peak value of salivary cortisol (e.g., sample 2, 3, 4, or 5) and the level of salivary cortisol at awakening (sample 1). The area under the response curve (AUCr) was measured together with single baseline levels of salivary cortisol, as mentioned elsewhere [17].

Data obtained from the participants were analyzed with the use of non-parametric Wilcoxon signed rank tests. Linear regression was found to be useful when studying the relationships between the variables. All statistical analyses were carried out according to Curran-Everett D and Benos’ statistics guidelines [38].

## 3. Results

Table 1 summarizes the results obtained in the current study concerning the mood measurements. The stress level of the samples varied significantly: the PSS ranged between 21 and 36 (mean value: 28.2 ± 5.94), the TMD fluctuated from 143 up to 291 (mean value: 186.7 ± 34.16), and the salivary cortisol, expressed as AUCr, had mean values from 20.19 up to 26.17 nmol/L (mean value: 23.0 nmol/L ±1.41). No significant differences were observed when comparing mean scores of right-handed subjects with those of left-handed participants for PSS, TMD and AUCr.

**Figure 2:**
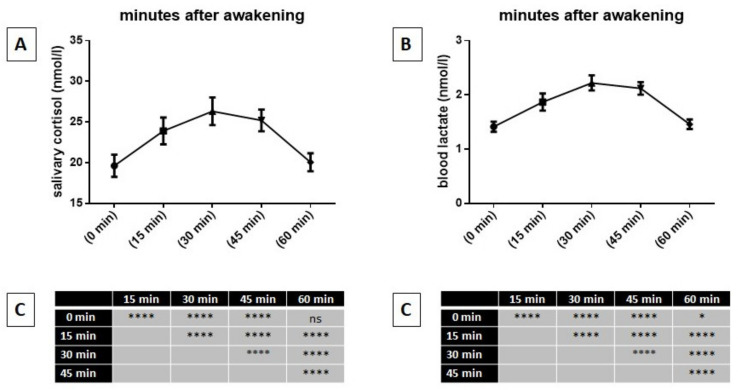
Mean values of profiles of salivary cortisol at awakening of the whole sample (**A**), as well as mean values of blood lactate levels at awakening of the whole sample (**B**) and results of Tukey’s multiple comparisons test between mean values (**C**). Abbreviations: ****, *p* > 0.0001; ns, not significant.

Figure 2 shows, on the left, the mean values of the awakening profiles of salivary cortisol for the total sample (A) and the results obtained with the Tukey’s multiple comparisons test after comparing them with the mean values (C). On the right of the figure, it also shows the mean values of the awakening profiles of blood lactate levels for the total sample (B), and the results obtained with the Tukey’s multiple comparisons test after comparing them to the mean values (C).

It can be seen that a significant increase in salivary cortisol after awakening (time 0) was observed, reaching its highest level after 30 min. However, an hour after awakening, levels of salivary cortisol were not significantly different from those at time 0. It is also possible to observe that blood lactate levels showed significant increases which mimicked the temporal trend of salivary cortisol.

**Figure 3:**
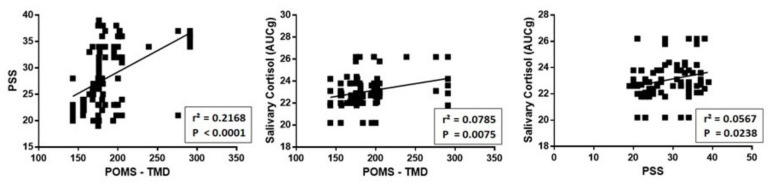
Relationship between POMS, expressed as TMD, PSS and level of salivary cortisol for each participant. As can be seen, there is a significant positive relationship between these three measures.

Figure 3 shows a highly significant positive relationship between POMS, expressed as TMD, PSS and salivary cortisol for each participant.

Regarding the postural control, Table 2 summarizes the results obtained when evaluating the A95 (in cm^2^). Comparing the data obtained from participants with open eyes, with that when their eyes were closed, it can be seen that in all positions, except the fourth, statistically significant differences were found. However, despite there being a statistical significance overall in P2, it can be observed that there was no statistically significant result obtained from left-handed participants in this position.

**Figure 4:**
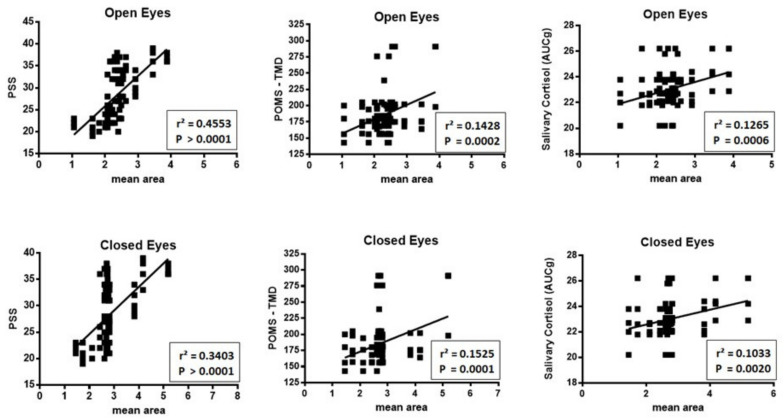
Relationship between scores, for each participant, of PSS, TMD and salivary cortisol with the mean value of A95 assessed in the five positions with open and close eyes. As can be seen, a significant correlation was observed for salivary cortisol, POMS and PSS with both open and closed eyes.

When the scores of PSS, TMD and salivary cortisol were compared with the mean value of A95 assessed in the five positions (Figure 4), a significant correlation was identified for salivary cortisol, POMS and PSS, with both open and closed eyes.

**Figure 5:**
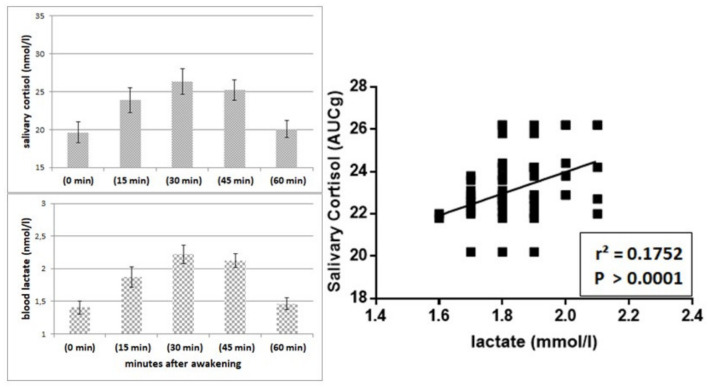
Relationship between salivary cortisol and blood lactate. On the left, the reader can observe the remarkable similarity of changes in salivary cortisol and blood lactate over the course of 60 min after awakening. On the right, however, one can appreciate the significantly positive linear relationship between the mean values of salivary cortisol and blood lactate in the 90 participants of the study.

Figure 5 shows the relationship between salivary cortisol and blood lactate. On the left, the reader can observe the remarkable similarity of changes in salivary cortisol and blood lactate over the course of 60 min after awakening. On the right, however, one can appreciate the significantly positive linear relationship between the mean values of these two variables in the 90 participants in the present study.

## 4. Discussion

The assessment of kinematic and kinetic variables has enabled the detection of cognitive influences on postural stability and gait [39,40,41,42]. In particular, it has been noted that high levels of stress can be a cofactor for less efficient abstract reasoning, executive function, visual-spatial memory, and processing speed [12,13,14]. Moreover, mood state seems to affect the control of balance [15] and anticipatory postural adjustments [16].

The main goal of the current study was to explore in young men: the levels of stress experienced; and their capability of maintaining an upright bipedal posture, while either having their eyes open or closed. The second goal was to analyze the possible influence of laterality.

The level of stress was evaluated with PSS, while general distress was measured with POMS expressed as TMD. Level of stress was also measured by assessing the rapid rise of salivary cortisol levels after awakening. Chronic stress seems to play a significant role in the CAR, but researchers investigating how chronic stress influences the CAR have described contradictory results (for reviews, see [21,22]). In fact, some authors observed a reduced CAR in relation with chronic stress, [43,44,45,46,47] whereas other authors showed an increased CAR in subjects continuously exposed to stressful conditions [48,49,50,51,52].

Stability of posture was inferred from A95, i.e., from the 95% of the area described by the CoP trajectory calculated while the subject maintained five different postures, each of which was maintained for at least 52 s, with and without visual input. The A95 parameter was chosen since preceding studies have showed that is the most sensitive measure for assessing postural stability [35,36]. Data about the CoP showed an significantly increased body sway in three specific conditions, i.e., when eyes were closed in the first position with the feet together (and therefore with the smallest support polygon) as well as in the fourth and fifth positions, where the dominant foot was 10 cm behind the other. The significant increase of body sway in the first position with closed eyes was not observed in a previous study on young women [17] and could represent a gender difference in postural control that should be studied further.

Moreover, it was observed that right-handed subjects exhibited a significant increase of body sway with closed eyes only in the fifth position, whereas this was displayed in left-handed participants only in the fourth position. Therefore, it seems possible to suppose that, when the two feet are not on the identical sagittal plane, it is easier to maintain a steady posture when the dominant foot is placed in front of the other, independent of visual input. This possible role for laterality strongly correlates with previous observations [17,53,54]. It could be suggested that the cerebellum plays a role in these postural differences in relation to laterality [55,56,57].

In the present study, a significant correlation was observed between PSS and TMD scores, as well as salivary cortisol and the mean value of A95 of CoP evaluated in the five positions when eyes were closed, corroborating what was previously observed in young women from using the same experimental protocol [17].

A final aspect we wanted to see in the present study was to see if the salivary cortisol increases in CAR were associated with rises of blood lactate levels. It has been observed that increases in salivary cortisol in CAR are associated with small but significant increases in blood lactate levels. This increase in blood lactate occurs in a condition where subjects were recently awakened but at physical rest. It follows that the cause of the blood lactate increase cannot be muscular but, presumably, of nervous origin. It is in fact well known that both neurons and astrocytes produce lactate, see [58] and, moreover, it has been observed that repetitive Transcranial Magnetic Stimulation in subjects completely at rest induces a significant increase in blood lactate [59].

## 5. Conclusions

In conclusion, the results of the present study confirm that the level of stress can influence postural stability, and that this influence is principally obvious when visual information cannot be used in postural control.

## Figures and Tables

**Table 1:** Salivary cortisol and mood.

Subject		PSS	POMS-TMD	Mean AUCg
All (N = 90)	mean	28.17	186.74	23.03
	SD	5.94	34.16	1.41
				
Right-handed (N = 71)	mean	28.26	181.32	22.60
	SD	6.09	32.07	1.23
				
Left-handed (N = 19)	mean	28.14	188.20	23.14
	SD	5.94	34.78	1.44

Scores obtained in the 90 participants with the Perceived Stress Scale (PSS; 10-item version) as well as with the Total Mood Disturbance (TMD) of Profile of Mood States (POMS). Mean values of salivary cortisol (nmol/L), expressed as area under the curve with respect to ground (AUCg), are also shown. Abbreviations: SD, standard deviation.

**Table 2:** Postural control of the sample.

Subjects		P1		P2		P3		P4		P5		Mean Value	
		OE	CE	OE	CE	OE	CE	OE	CE	OE	CE	OE	CE
All (N = 90)	mean	2.62	3.07	2.18	2.36	1.87	1.87	2.72	3.28	2.79	3.81	2.34	2.79
	SD	1.12	1.35	0.66	0.76	0.56	0.76	0.94	1.14	1.01	1.66	0.58	0.78
	W-test	*p* < 0.0001		*p* < 0.01		NS		*p* < 0.0001		*p* < 0.0001		*p* < 0.0001	
Right-handed (N = 71)	mean	2.67	3.13	2.22	2.37	1.84	1.83	2.72	3.12	2.80	3.96	2.41	2.88
	SD	1.08	1.30	0.69	0.78	0.57	0.79	0.96	1.09	1.08	1.76	0.67	0.92
	W-test	*p* < 0.0001		*p* < 0.05		NS		*p* < 0.001		*p* < 0.0001		*p* < 0.0001	
Left-handed (N = 19)	mean	2.41	2.87	2.06	2.32	1.99	2.02	2.72	3.88	2.78	3.23	2.28	2.77
	SD	1.26	1.56	0.52	0.70	0.53	0.62	0.86	1.12	0.73	1.10	0.63	0.85
	W-test	*p* < 0.01		NS		NS		*p* < 0.001		*p* < 0.05		*p* < 0.0001	

Area of Center of Pressure expressed as A95 (95% confidence ellipse expressed in cm^2^) measured in the 90 participants in the five different positions shown in Figure 1. Each position was maintained for at least for 52 s with open eyes (OE) and closed eyes (CE). Note the difference between right-handed and left-handed subjects. Abbreviations: SD, standard deviation; NS, not significant; W-test, Wilcoxon signed rank test.
